# Therapeutic Potential of Plant Oxylipins

**DOI:** 10.3390/ijms232314627

**Published:** 2022-11-23

**Authors:** Tatyana Savchenko, Evgeny Degtyaryov, Yaroslav Radzyukevich, Vlada Buryak

**Affiliations:** 1 Institute of Basic Biological Problems, Pushchino Scientific Center for Biological Research, Russian Academy of Sciences, 142290 Pushchino, Russia; 2 Puschchino State Institute of Natural Sciences, Prospect Nauki st., 3, 142290 Pushchino, Russia; 3 Faculty of Biotechnology, Moscow State University, Leninskie Gory 1, str. 51, 119991 Moscow, Russia; 4 Branch of Shemyakin and Ovchinnikov Institute of Bioorganic Chemistry, Russian Academy of Sciences, 142290 Pushchino, Russia

**Keywords:** plant oxylipins, acetylenic oxylipins, jasmonates, traumatic acid, phytoprostanes and phytofurans, oxy-, hydroxy-, and epoxy-derivatives of fatty acids

## Abstract

For immobile plants, the main means of protection against adverse environmental factors is the biosynthesis of various secondary (specialized) metabolites. The extreme diversity and high biological activity of these metabolites determine the researchers’ interest in plants as a source of therapeutic agents. Oxylipins, oxygenated derivatives of fatty acids, are particularly promising in this regard. Plant oxylipins, which are characterized by a diversity of chemical structures, can exert protective and therapeutic properties in animal cells. While the therapeutic potential of some classes of plant oxylipins, such as jasmonates and acetylenic oxylipins, has been analyzed thoroughly, other oxylipins are barely studied in this regard. Here, we present a comprehensive overview of the therapeutic potential of all major classes of plant oxylipins, including derivatives of acetylenic fatty acids, jasmonates, six- and nine-carbon aldehydes, oxy-, epoxy-, and hydroxy-derivatives of fatty acids, as well as spontaneously formed phytoprostanes and phytofurans. The presented analysis will provide an impetus for further research investigating the beneficial properties of these secondary metabolites and bringing them closer to practical applications.

## 1. Introduction

The list of drugs generated from plant metabolites or their synthetic analogs includes antibacterial, antifungal, antiviral, antiparasitic, anticancer, antidiabetic, anti-inflammatory, and other agents [1,2,3,4]. Notable examples are apomorphine (Apokyn^®^) used to treat Parkinson’s disease, nitisinone (Orphadin^®^) used as a treatment for hereditary tyrosinemia, and miglustat (Zavesca^®^) prescribed for the treatment of Gaucher disease [5]. Two of the most important anti-cancer drugs—taxol (isolated from *Taxus brevifolia* L.) and camptothecin (from *Camptotheca acuminate*)—were selected in the so-called “random screening” of plant metabolites [6]. Currently, a significant part of the pharmaceutical industry’s income depends on plant metabolites. The use of plant material as a source of valuable metabolites has great economic importance since the global biomedical materials market is projected to reach US$232,280 million by 2028, up from US$110,240 million in 2021 [1,7].

The ability of immobile plants to produce various secondary (specialized) metabolites with high biological activity is associated with the need to adapt to a constantly changing environment. An important class of secondary metabolites used by plants for protection against adverse environmental factors is represented by oxylipins. It is known that plant lipids and free fatty acids, the substrates for oxylipin biosynthesis, can modulate the physiological processes in the animal cell, for example, by exhibiting immunomodulatory properties [8], inducing apoptosis in cancer cells [9], or inhibiting expression of pro-inflammatory cytokines [10]. The significant diversity and functional activity of oxylipins make their therapeutic and pharmaceutical potential even greater than that of fatty acids and lipids. The ability of plant oxylipins to influence processes in a non-plant cell can also be explained by the fact that the oxylipin biosynthesis and signaling pathways have significant similarities in evolutionarily distant species. There are similarities between plants and animals in the biochemical nature of the oxylipin biosynthesis enzymes and the chemical structure of some oxylipins. The functional similarity is also obvious since these metabolites are involved in stress responses in both plants and animals [11]. In animal cells, biologically active eicosanoids, such as prostaglandins, prostacyclins, thromboxanes, leukotrienes, lipoxins, and others, are formed from 20-carbon arachidonic acid [12,13]. These oxylipins perform important regulatory functions in all tissues and systems of the animal body and are involved in various immunopathological processes, including inflammation, autoimmune responses, and cancer [13,14,15]. Plant oxylipins are involved in defense responses under abiotic (sub-optimal temperatures, drought, UV radiation, etc.) and biotic (bacterial and fungal infections, viruses, attacks by herbivorous insects and animals) stresses [16,17,18]. Fatty acids and oxylipins are also involved in inter-organismal signaling functions. Thus, eicosapolyenoic fatty acids, which enter plant tissues upon infection with oomycete pathogens, can modify plant defense responses [19], and Arabidopsis transgenic plants, accumulate 20-carbon fatty acids unusual for plant vegetative tissues, exhibit altered responses to environmental stimuli [11]. Some volatile oxylipins act as mobile signals in plant-plant and plant-insect interactions [20,21].

Although the beneficial properties of the representatives of individual classes of plant oxylipins have been uncovered, the therapeutic potential of this big group of secondary metabolites is far from being revealed. Here we present an analysis of available data on the therapeutic potential of all major classes of plant oxylipins, including derivatives of acetylenic fatty acids, jasmonates, hydroperoxide lyase branch-produced oxylipins, oxy-, epoxy-, and hydroxy-derivatives of fatty acids, as well as spontaneously formed phytoprostanes and phytofurans.

## 2. Variety of Plant Oxylipins

**Figure 1:**
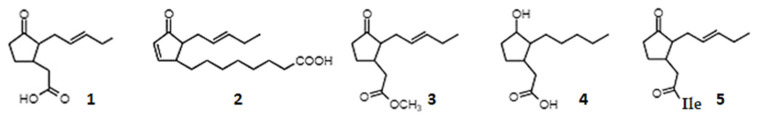
Chemical structures of several jasmonates: JA, (3-oxo-2-(2-pentenyl) cyclopentaneacetic acid (**1**); 12-oxo-phytodienoic acid (**2**); MeJA (**3**); (3-hydroxy-2-pentylcyclopentyl)-acetic acid (**4**); and jasmonoyl-L-isoleucine (**5**).

Oxylipins are formed enzymatically or spontaneously from fatty acids in all aerobic organisms, from bacteria to humans [22]. In higher plants, 16- and 18-carbon unsaturated fatty acids are the main substrates for oxylipin biosynthesis [11,23]. Plant oxylipins are comprised of fatty acid hydroperoxides, cyclopentenone compounds, aldehydes, ketoacids, divinyl ethers, epoxides, epoxy alcohols, and others [16,24,25,26,27,28,29] (Figure 1). A significant contribution to the functional diversity is also made by the presence of oxylipins in plant tissue, in free form and as conjugates with amino acids, carbohydrates, glutathione, ethanolamine, lipids, and other compounds [30]. The enzymatic formation of oxylipins in plants is mainly associated with the activity of the lipoxygenase (LOX) pathway, which begins with the regio- and stereospecific dioxygenation of polyunsaturated fatty acids by 9- and 13-specific lipoxygenases, non-heme iron-containing dioxygenases [31,32]. In lower photosynthetic organisms, hydroperoxides of twenty-carbon fatty acids can also be formed [33]. Interestingly, lipoxygenases can oxidize both free and lipid-bound fatty acids [32,34,35].

In addition to LOX-dependent oxylipin biosynthesis pathways, there is an alternative α-dioxygenase (α-DOG) pathway of fatty acid oxidation. α-DOGs are heme-containing proteins in which the prosthetic group can be linked both covalently and non-covalently to the polypeptide. α-Oxygenation catalyzed by α-DOG, as in the case of LOX, leads to the formation of hydroperoxy-derivatives of fatty acids, but this reaction occurs exclusively on the α-carbon of the carbon chain. The unstable hydroperoxy derivative can be further converted to the corresponding hydroxy derivative or truncated (17-carbon) aldehyde or acid [36].

The 9- and 13-hydroperoxides of fatty acids formed by lipoxygenases can be further modified in several branches of the oxylipin biosynthesis pathway, initiated by one of the following enzymes: allene oxide synthase (AOS), hydroperoxide lyase (HPL), divinyl ether synthase (DES), peroxygenase, epoxy alcohol synthase (EAS), and reductase [16,26,37,38,39]. Lipoxygenases can also implement the secondary oxidation of hydroperoxides to form keto derivatives [40]. Three of these enzymes (AOS, HPL, and DES) belong to the CYP74 family of the cytochrome P450-dependent monooxygenase superfamily. Unlike other cytochrome P450 superfamily members, CYP74 enzymes do not require NADPH for enzymatic activity and use fatty acid hydroperoxides simultaneously as a substrate and as an oxygen donor [18,22,41,42]. Known epoxy alcohol synthases mostly belong to the CYP74 clan (share less than 40% sequence identity with other CYP74) and, in rare cases, to the CYP74 family [43,44]. Peroxygenases (lipid peroxygenases) are calcium-binding proteins and heme-containing oxygenases, which catalyze the oxidation of hydroperoxides of unsaturated fatty acids with the formation of epoxy-, hydroxy-, and epoxyhydroxy derivatives. Peroxygenases are not related to peroxidases. They also do not belong to the CYP74 family but belong to a small protein family called caleosin [45,46,47].

The allene oxide synthase and hydroperoxide lyase branches are the dominant enzymatic pathways for the biosynthesis of oxylipins in most higher plants. In the allene oxide synthase branch, so-called jasmonates are formed, which structurally resemble animal eicosanoids (Figure 1). Jasmonates, present in all land plants, are the most studied group of oxylipins. These oxylipins perform the functions of phytohormones that regulate plant growth, development, and defense responses. The main metabolites of this branch are jasmonic acid (JA, 3-oxo-2-(2-Z)-2-pentenylcyclopentaneacetic acid) (**1**), its methyl derivative—methyl jasmonate (MeJA) (**3**) [48], its biosynthetic precursors of jasmonic acid—12-oxo-phytodienoic acid (12-OPDA) (**2**), and dinor-12-oxophytodienoic acid (dn-OPDA) [49,50], as well as conjugates of jasmonates with amino acids, primarily with isoleucine (**5**) [51,52,53]. In plant tissues, glucosylated, carboxylated, hydroxylated, and other jasmonate derivatives are present, however, the formation of most of these compounds is a mechanism for removing active jasmonates from cells [54,55,56,57]. In the plant cell, there is a sophisticated signaling system based on jasmonates that carries out highly specific regulation of hundreds of jasmonate-dependent genes. The main signaling molecule in this system is the conjugate of jasmonic acid with isoleucine (jasmonoyl-L-isoleucine) (**5**) [58,59].

**Figure 2:**
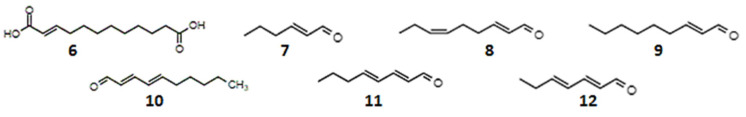
Chemical structures of hydroperoxide lyase branch oxylipins: traumatic acid (**6**); (E)-2-hexenal (**7**); (2E,6Z)-2,6-nonadienal (**8**); (E)-2-nonenal (**9**); (2E,4E)-2,4-decadienal (**10**); (2E,4E)-2,4-octadienal (**11**); and (2E,4E)-2,4-heptadienal (**12**).

HPL is present in many, but not all plants, where plants may contain one or more enzymes that differ in substrate specificity and intracellular localization [60]. With the participation of 13-HPL, 6-carbon aldehydes and 12-carbon aldoacids are formed from 13-hydroperoxides of 18-carbon fatty acids (Figure 2). The unstable 12-carbon compound of the HPL branch, 12-oxo-9(Z)-dodecenoic acid, isomerizes to the more stable 12-oxo-10(E)-dodecenoic acid, referred to as traumatin or wound hormone. Oxidation of the aldehyde group of traumatin leads to the formation of 2(E)-dodecene-1,12-dicarboxylic acid (traumatic acid) (**6**) [61,62]. Several isomers of traumatin and traumatic acid are present in plant tissues. 9-Specific HPLs use 9-hydroperoxides of linoleic and linolenic acids to form two 9-carbon compounds, the volatile products nonenal (**9**) and nonadienal (**8**), respectively, and the less volatile 9-oxonanoic acid. The product formation in an HPL-catalyzed reaction occurs through the formation of an unstable hemiacetal intermediate [63,64]. Subsequently, aldehydes can isomerize and turn into alcohols, hydroxy, and aceto derivatives. These volatile aldehydes and their derivatives, collectively known as Green Leaf Volatiles, are the main component of the aroma of green leaves and fruits; they protect plants from insects and pathogens and mediate interactions with other organisms [65].

**Figure 3:**
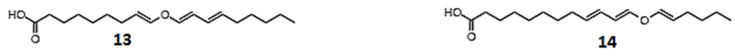
Chemical structures of plant oxylipins, divinyl ether fatty acids: colneleic acid (**13**) and etheroleic acid (**14**).

Divinyl ether synthases, which are less common in plants, use 9- or 13-hydroperoxides of fatty acids, primarily linoleic and α-linolenic acids, to form divinyl ether fatty acids (Figure 3), such as colnelic, colneleic (**13**), etheroleic (**14**), and etherolenic acids, as well as their numerous isomers [26,66]. Known divinyl ether synthases differ in substrate specificity: DES from tomato, tobacco, and potato primarily use 9-hydroperoxide of fatty acids [29,67], whereas DES from garlic use 13-hydroperoxide of fatty acids [68].

The existence of CYP74 enzymes with dual activity, such as those with simultaneous hydroperoxide lyase and epoxyalcohol synthase activity (9/13-HPL/EAS), is extremely intriguing [69]. This dual product specificity, also observed in several AOSs and DESs, seems to expand the biological functions of oxylipin biosynthesis enzymes [69,70,71]. Such enzymatic plasticity is also a valuable property for biotechnological applications.

**Figure 4:**
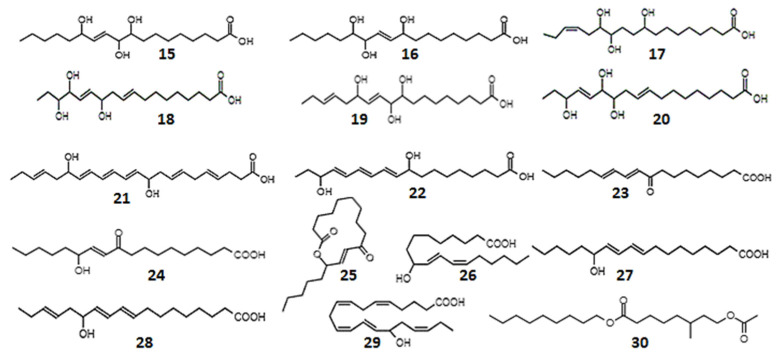
Chemical structures of plant oxylipins, oxy-, hydroxy-, and epoxy-derivatives of fatty acids: 9,10,13-trihydroxy-11-octadecenoic (**15**); 9,12,13-trihydroxy-l0-octadecenoic acid (**16**); 9,12,13-trihydroxy-15-octadecadienoic (**17**); 12,15,16-trihydroxy-9,13-octadecadienoic (**18**); 9,10,13-trihydroxy-11,15-octadecadienoic (**19**); 12,13,16-trihydroxy-9,14-octadecadienoic acid (**20**); 10,17-dihydroxy-docosahexa-4,7,11,13,15,19-enoic acid (**21**); 9,16-dihydroxy-10,12,14-octadecatrienoic acid, isomers (**22**); 9-oxo-10,12-octadecadienoic acid (**23**); 13-hydroxy-10-oxo-11-octadecenoic acid (**24**); 10-oxo-11-octadecen-13-olide, en-antiomers (**25**); 9-hydroxy-10,12-octadecadienoic acid (**26**); 13-hydroxy-9,11-octadecadienoic acid (**27**); 13-hydroxyoctadeca-9,11,15-trienoic acid (**28**); 15-hydroxyeicosa-5,8,11,13,17-pentaenoic acid (**29**); and nonyl 8-acetoxy-6-methyloctanoate (**30**).

EAS converts fatty acid hydroperoxides to epoxy alcohols, which can be further converted to epoxyhydroxy and hydroxy derivatives (Figure 4) [72,73]. The epoxy alcohols can also be formed by peroxygenases [74,75], as well as in non-enzymatic and pseudo-enzymatic reactions in the presence of transition metals and hemoproteins [73,76].

Peroxygenases (caleosin/peroxygenase proteins) associate with lipid membranes or lipid inclusions and form several products, including epoxy, epoxyhydroxy, and hydroxy derivatives (Figure 4) [77]. Unlike epoxygenases, which can also form epoxides, peroxygenases do not require NADPH for the enzymatic reaction. The products of the peroxygenase reaction are similar to the products of the epoxyalcohol synthase reaction [78]. Subsequent transformation of epoxy derivatives by epoxide hydrolases leads to the formation of trihydroxy acids [45,46,79].

**Figure 5:**
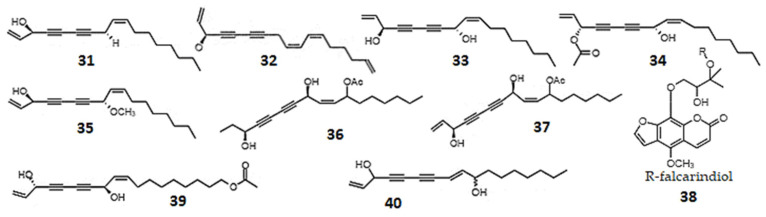
Chemical structures of plant oxylipins, derivatives of acetylenic fatty acids: falcarinol (**31**); didehydrofalcarinol (**32**); falcarindiol (**33**); falcarindiol-3-acetate (**34**); falcarindiol-8-methyl ether (**35**); 1,2-dihydro-11-acetoxy-falcarindiol (**36**); 11-acetoxy-falcarindiol (**37**); furanocoumarin ethers of falcarindiol, where R indicates falcarindiol (**38**); 11(*S*),16(*R*)-dihydroxy-octadeca-9Z,17-dien-12,14-diyn-1-yl acetate (**39**); and panaxydiol (**40**).

An interesting class of oxylipins is represented by the derivatives of fatty acids containing triple bonds (Figure 5). Fatty acids containing one triple bond (acetylenic fatty acids) or several triple bonds (polyacetylenic fatty acids) are quite widespread and are found in algae, mosses, lichens, and higher plants, although in small amounts in most cases [80,81]. The triple bonds present in the molecules determine the high chemical activity of these oxylipins and, as a result, their high biological activity. That is why the derivatives of acetylenic fatty acids are studied more than other plant oxylipins in terms of their effect on the animal cell, which is reflected in several review articles [80,81,82,83,84]. These compounds act as alkylating agents, capable of modifying various molecules, including proteins, so high concentrations of acetylenic fatty acid derivatives are toxic [85]. The high lipophilicity of these oxylipins increases their ability to permeate the cell membranes.

**Figure 6:**
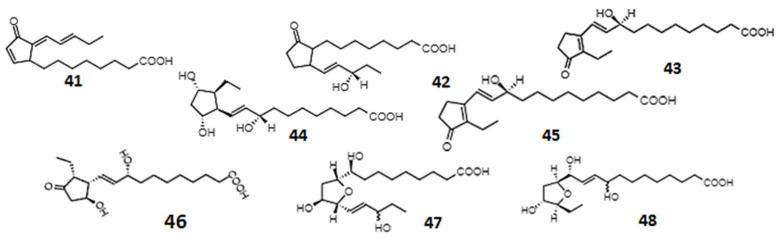
Chemical structures of phytoprostanes and phytofurans: deoxy-J1-PhytoP (**41**); 16-B1-PhytoP (**42**); 9-L1-PhytoP (**43**); 9-*epi*-9-F1t-PhytoP (**44**); ent-16-epi-16-F1t-PhytoP (**45**); 9-epi-9-D1t-PhytoP (**46**); ent-16(RS)-9-epi-STΔ14-13-PhytoF (**47**); and ent-9(RS)-12-epi-ST-9-Δ10-13-PhytoF (**48**).

Spontaneous oxidation of fatty acids leads to the formation of phytoprostanes (Figure 6), compounds with structural similarity to isoprostanes and prostanoids—the powerful regulators of physiological responses in animal cells formed from arachidonic acid [86,87,88,89,90]. Phytoprostanes (PhytoPs) are formed from polyunsaturated fatty acids in both photosynthetic and non-photosynthetic plant tissues, although their total content in photosynthetic tissues is ten times higher [91]. Phytoprostanes, similarly to animal isoprostanes, can be formed from free fatty acids and fatty acids bound to lipids and later released by lipases [92]. The spontaneous oxidation of fatty acids initially leads to the formation of hydroperoxides and cyclic peroxides. Depending on the number of carbon atoms in the fatty acid chain (C-14 or C-11), which loses hydrogen and then adds oxygen, two types of phytoprostanes G1 (PPG1-phytoprostanes G1), type 1 and type 2, respectively, are formed, where each type includes 16 isomers. PPG1s are spontaneously reorganized or reduced to form cyclic compounds PPD1, PPE1, and PPF1, and finally, dehydration and isomerization of PPD1 and PPE1 result in the formation of PPJ1, deoxy-PPJ1 (**41**), PPA1, and PPB1 [86,88]. The presence of racemic regioisomers increases the diversity of phytoprostanes in the plant cell. The most commonly encountered phytoprostanes are PPE1 and PPF1. At high concentrations, oxygen reacts with the endoperoxide carbon radical to generate tetrahydrofuran ring-containing compounds. Thus, phytoprostanes are converted into phytofurans [93]. The biological activity of many phytoprostanes and phytofurans is very high. They have some, although not all, properties of the plant hormone jasmonates, including the ability to activate the biosynthesis of secondary metabolites and induce the expression of genes involved in detoxification processes, and at the same time perform functions that are not typical for jasmonates [87,94,95].

Some oxylipins, such as phytoprostanes, 12-OPDA (**2**), acrolein, 2-hexenal (**7**), and others, are classified as so-called reactive electrophile species (RES), which are characterized by the presence of α,β-unsaturated carbonyl group. In these compounds, the proximity of the double bond increases the electrophilicity of the carbonyl group and enables the interaction with the nucleophilic regions of various organic molecules, such as glutathione, proteins, and nucleic acids [96]. This binding changes the properties of the target molecules.

Thus, the significant structural diversity of plant oxylipins in combination with their high reactivity points to the significant potential of these compounds for a practical application not only as biocontrol agents in agriculture [97] but also in medicine.

## 3. Potential Cellular Targets for Inflammation, Allergy, and Cancer Treatment

With all the variety of intracellular processes responsible for the development of various diseases, a significant portion of pathological reactions are associated with inflammation, allergy, and malignant cellular transformations. In this chapter, we present a brief description of the molecular processes involved in inflammation, allergy, or cancer, which may serve as targets for plant oxylipins according to the available modern literature data.

Inflammation is the normal biological response of the body to physical, chemical, or biological stimuli [98]. In some pathological conditions, chronic inflammation can lead to the development of various diseases, such as rheumatoid arthritis, asthma, and type 2 diabetes. Sometimes chronic inflammation stimulates cancer progression. Pro-inflammatory cytokines (interleukins IL-1, IL-6, IL-8, and tumor necrosis factor TNF-α), NO, platelet-activating factor, histamine, and other inflammatory mediators can significantly contribute to the development of inflammation [99]. The secretion of inflammatory mediators causes an increase in vascular permeability and a deceleration of blood flow, which leads to the recruitment of leukocytes. Leukocytes secrete cytokines that promote the secretion of other inflammatory mediators and attract macrophages to the site of inflammation, thereby enhancing the inflammatory process [100].

**Figure 7:**
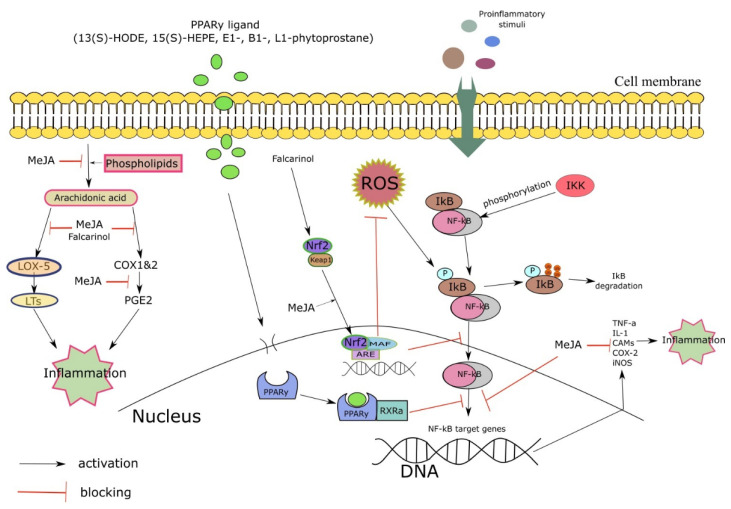
Schematic representation of the molecular mechanisms underlying the ability of plant oxylipins to exhibit anti-inflammatory and antioxidant activity. The internal or external proinflammatory ligands activate several intracellular pathways leading to the synthesis of proinflammatory cytokines. Plant oxylipins can affect inflammation by activating nuclear factors (NF-kB, Nrf2, PPAR) or suppressing ROS formation and COX/LOX activity.

The biological effects of various classes of oxylipins are based on their ability to modulate the inflammation-associated intracellular signaling pathways in animal cells, thereby regulating the expression of pro-inflammatory mediators. The spectrum of intracellular signaling pathways regulated by oxylipins is broad: from protein kinase c-beta (PKC-β) to nuclear factor NF-κB and peroxisome proliferation activator receptor (PPAR) (Figure 7). Due to a significant number of regulated signaling pathways, plant oxylipins show a wide range of biological activities, from suppression of the inflammatory response to regulation of the cell cycle and apoptosis, however, the listed intracellular pathways are the most frequent targets for plant oxylipins [101].

### 3.1. Nuclear Factor NF-κB

Transcription factor NF-κB regulates many aspects of innate and adaptive immunity and serves as a major mediator of inflammatory responses. The NF-κB family includes NF-κB1 (p50/p105), NF-κB2 (p52/p100), p65 (RelA), RelB, and c-Rel [102]. NF-κB induces the expression of various pro-inflammatory cytokines (IL-6, IL-8, and TNF-α) and is involved in the regulation of inflammation. In addition, NF-κB plays a critical role in regulating the survival, activation, and differentiation of innate immune cells [103]. Uncontrolled NF-κB activation leads to the pathogenic processes of various inflammatory diseases. NF-κB is normally present in an inactive form in the cytoplasm in association with inhibitory IκB proteins [104]. Upon activation of the signaling pathway, inhibitory proteins get phosphorylated and release NF-κB.

Since uncontrolled NF-κB activation is associated with various inflammatory diseases, targeting the NF-κB signaling pathway represents a promising approach for anti-inflammatory therapy. However, despite significant progress in developing approaches to inhibit NF-κB, there are challenges in developing clinically available drugs. The major concern is the balance between inhibition efficiency and safety since the activity of this transcription factor is also required for the maintenance of a normal immune response and cell survival. Accumulated research data show that complete inhibition of NF-κB signaling can cause serious side effects [105]. Several oxylipins, phytoprostanes, acetylenic oxylipins, methyl jasmonate, and others can affect this nuclear factor.

### 3.2. Peroxisome Proliferator-Activated Receptor (PPAR)

The key regulators of lipid metabolism are peroxisome proliferator-activated receptors (PPARs). These receptors exist in three different isoforms: PPARα (NR1C1), PPARβ/δ (NR1C2), and PPARγ (NR1C3). They are synthesized mainly in adipose tissue, activated by fatty acids and their derivatives, and serve as the so-called lipid sensors of the body, regulating the metabolism of carbohydrates and lipids. PPARs belong to a group of nuclear receptors. They heterodimerize with retinoic X receptors (RXR) and, upon ligand binding, act primarily as transcriptional regulators of specific target genes. Depending on tissue, cofactors, and ligand availability, PPARs perform multiple functions [106]. PPARγ is a type II nuclear receptor that functions as a transcription factor [107]. Many naturally occurring agents bind directly to PPARγ and activate it. These include the 15-hydroxyeicosatetraenoic acid [108], the phytocannabinoid—tetrahydrocannabinol [109], and its synthetic analog [110]. PPAR activity is also affected by phytoprostanes and 13-hydroxyoctadecadienoic acid (**27**). Activation of PPARγ by these and other ligands may be responsible for the growth inhibition of cultured breast, gastric, lung, prostate, and other cancer cell lines. PPARγ is implicated in the pathology of many diseases, including obesity, diabetes, atherosclerosis, and cancer [111].

### 3.3. Other Cellular Targets

In addition to PPAR and NF-κB, oxylipins can influence PKCβ (protein kinase C-β), a protein involved in many cellular signaling pathways and regulating various cellular functions such as B cell activation, apoptosis induction, and endothelial cell proliferation [112].

Acetylenic oxylipins may interfere with the Keap1-Nrf2 pathway involved in the detoxification of carcinogenic agents [80]. In addition, they have an inhibitory effect on 5-, 12-, and 15-lipoxygenases and cyclooxygenases, which are involved in tumorigenesis [113].

The system, including kelch-like ECH-associated protein 1 (KEAP1)/nuclear factor associated with erythroid factor 2 (NRF2), is one of the most important cellular defense systems and survival pathways in vivo. NRF2 is anchored in the cytoplasm by KEAP1 at rest and translocated to the nucleus to activate the antioxidant response element (ARE) under conditions of oxidative stress, which in turn leads to the increased expression of antioxidant proteins. However, it has been reported that NRF2 protects not only normal cells but also tumor cells from oxidative damage [114].

Lipoxygenase and cyclooxygenase pathways are associated with oxylipin synthesis, including prostanoids and leukotrienes [115]. Prostanoids control a wide range of biological processes, from blood pressure homeostasis and inflammation to cell survival. Disruption of normal prostanoid signaling is associated with numerous diseases. Prostanoids also modulate neuronal activity by inhibiting or stimulating the release of neurotransmitters, sensitizing sensory nerve fibers to harmful stimuli, or inducing fever or sleep. They are involved in apoptosis, cell differentiation, and oncogenesis [116]. Leukotrienes are lipid-derived mediators that play a key role in acute and chronic inflammation and various allergic diseases, including asthma (neutrophilic asthma and aspirin-sensitive asthma), allergic rhinitis, atopic dermatitis, allergic conjunctivitis, and anaphylaxis [117].

## 4. Therapeutic Properties of Plant Oxylipins

### 4.1. Acetylenic Fatty Acid Derivatives

Derivatives of acetylenic fatty acids from various plant species, including representatives of well-known medicinal plants of the Apiaceae, Araliaceae, and Asteraceae families, and synthetic analogs of these plant metabolites exhibit useful properties, primarily as antimicrobial and anticancer compounds [80,81]. Thus, falcarindiol (**33**) and falcarinol (**31**) have been shown to have antifungal activity by inhibiting the formation of fungal spores [82,118]. These oxylipins also exhibit antibacterial activity by inhibiting the growth of mycobacteria *Mycobacterium* ssp., [119,120], gram-positive bacteria *Bacillus subtilis,* and staphylococcus *Staphylococcus aureus* at safe concentration for human health of 10 μg/mL [121]. Falcarindiol (**33**) strongly inhibited the growth of *Micrococcus luteus* and *Bacillus cereus* in vitro with a minimal inhibitory concentration of 50 μg/mL [122]. In vitro activity of (3S)-16,17-didehydrofalcarinol (**32**) isolated from *Tridax procumbens* against *Leishmania mexicana*, a protozoan causing cutaneous leishmaniasis, has been shown [123]. This oxylipin exerts a direct inhibitory effect on the parasite at the intracellular stage (amastigote) without any negative effect on the host cells. Presumably, the observed antiamastigotic activity is not associated with known defense mechanisms based on the activation of NO-mediated responses in macrophages.

The anticancer properties of polyacetylenic oxylipins have been extensively studied. Antitumor activity has been shown for falcarinol (**31**) and related compounds such as falcarindiol-8-methyl ether (**35**), panaxydiol (**40**), and panaxitriol from plants of the Apiaceae, Araliaceae, and Asteraceae families [80,124]. These compounds have a pronounced cytotoxic effect on cancer cell lines, specifically inducing cell cycle arrest and apoptosis in cancer cells. At the same time, they have a chemoprotective effect on healthy cells due to their ability to suppress the synthesis of pro-inflammatory proteins and induce “endoplasmic reticulum stress” [80]. Antiproliferative activity has also been demonstrated for furanocoumarin ethers of falcarindiol (**38**) [125]. (3S)-16,17-Didehydrofalcarinol (**32**) has been shown to inhibit colon cancer cell proliferation [126]. C17 acetylenic oxylipins from *Eryngium tricuspidatum*, including two rare oxylipins, 11-acetoxy-falcarindiol (**37**) and 1,2-dihydro-11-acetoxy-falcarindiol (**36**), inhibited all cancer cell lines tested in vitro at concentrations ranging from 0.3–29 μM [127]. Morphological assessment of these oxylipins’ effect on SKMEL-28 melanoma cells using video-enhanced phase-contrast microscopy suggested a similar mechanism of apoptosis induction to that observed upon falcarindiol (**33**) treatment of colon cancer [127].

The high anticancer activity of acetylenic oxylipins is stipulated by several characteristic features of their chemical structure. Besides the chemical activity associated with the presence of triple bonds, the acetyl group of these oxylipins interacts with the thiol group of cysteine, which in turn affects the Keap1-Nrf2 pathway involved in the detoxification of carcinogenic agents and the formation of anti-inflammatory cytokines. Moreover, acetylenic oxylipins, in particular oxylipins from plants of the Apiaceae, Araliaceae, and Asteraceae families, can be a ligand for the nuclear receptor PPARγ, which performs important functions in the regulation of cancer cell growth, proliferation, differentiation, apoptosis, and also the metabolism of fatty acids and carbohydrates [80]. Falcarindiol (**33**) and compound 11(*S*),16(*R*)-dihydroxy-octadeca-9Z,17-dien-12,14-diyn-1-yl acetate (**39**) from Apiaceae and Araliaceae exert an inhibitory effect on 5-, 12-, and 15-lipoxygenases and cyclooxygenases, which are involved in tumorigenesis, at fairly low concentrations (IC50 values of 73 μM and 24 μM, respectively) [83,113,128,129,130].

Acetylenic oxylipins can affect inflammation. Falcarinol (**31**), falcarindiol (**33**), and falcarindiol-3-acetate (**34**) are responsible for the anti-inflammatory properties of purple carrots [131]. These acetylenic oxylipins inhibit the NF-κB pathway, a key pathway regulating the expression of genes involved in pro-inflammatory processes.

The antiplatelet effect of falcarinol and falcarindiol is most likely also related to their anti-inflammatory activity and their ability to regulate lipoxygenases responsible for the formation of thromboxanes, in particular thromboxanes B2 [113,132]. The antiplatelet properties of falcarinol and panaxynol may also be associated with the ability to inhibit the enzyme 15-hydroxyprostaglandin dehydrogenase, responsible for the catabolism of prostaglandins [133]. The ability of falcarinol and falcarindiol to prevent the development of atherosclerosis is also associated with the inhibition of 5-, 12-, and 15-lipoxygenases [129,130]. Thus, the anti-inflammatory, anticoagulant, and partly anticancer properties of oxidized derivatives of acetylenic fatty acids can also be associated with their alkylating abilities, as well as with their ability to inhibit the lipoxygenase, cyclooxygenase, and NF-κB pathways.

In addition to the described activities, falcarinol has been shown to have neuroprotective properties, which can be used in the treatment of neurodegenerative diseases such as Alzheimer’s disease [134,135]. The neuroprotective properties of falkarinol were shown to be associated with the ability to affect paraneurons, the cells of epithelial origin that are not nervous but can generate an action potential, secrete neurotransmitters, and stimulate neuritogenesis (neurite formation). The stimulation of neuritogenesis is most likely also responsible for the positive effect of falcarinol on memory in mice after exposure to scopolamine, inducing significant memory impairment in rodents [134].

The compounds 11(*S*),16(*R*)-dihydroxy-octadeca-9Z,17-dien-12,14-diyn-1-yl acetate (**39**), and (3R,8S)-falcarindiol (**33**) are thought to be responsible for the medicinal properties of the ginseng plant *Angelica sinensis* (Oliv.) Diels (Apiaceae), which is known for its beneficial effect on women’s health, and emotional state during the premenstrual period and menopause. The ability of these compounds to bind to the serotonin receptor 5-HT7 has been confirmed [136]. Serotonin is often called the “good mood hormone” and “happiness hormone”. Therefore recently, this neurotransmitter has been considered a target for the development of new drugs for the treatment of various diseases, in particular, antidepressants.

Several medical drugs inhibiting calcium signal transduction pathways used in the treatment of dementia, allergies, cancer, angina pectoris, and diabetes have been created based on acetylenic oxylipins [137]. A drug containing falcarinol is used in the treatment of cardiovascular, inflammatory, neurodegenerative, and viral diseases, as well as cancer and liver diseases [138]. The anti-inflammatory and bactericidal activities of polyacetylene oxylipins and their ability to inhibit cyclooxygenase and lipoxygenase enzymes are described in several patents [139,140,141].

### 4.2. Jasmonates

Jasmonates are widespread in the plant kingdom. These hormones regulate plant growth, development, and the formation of defense mechanisms in adverse environmental conditions [39]. The ability of these active compounds to influence the biochemical processes in the animal cell has been demonstrated in many studies. Jasmonates and their derivatives have both indirect and direct effects on human health. The indirect effect is associated with the stimulation of the synthesis of compounds with health-beneficial properties [39,142]. One of many examples is the application of methyl jasmonate (MeJA) on fruit crops (strawberries, raspberries, blackberries, grapes, and apples) to increase the content of antioxidant compounds and various phenolic metabolites, including anthocyanins. Anthocyanins, flavonoids, and phenolic acid derivatives are highly effective in inhibiting the oxidation of low-density lipoproteins in humans. Epidemiological studies show that a human diet rich in natural plant-derived polyphenols can reduce the risk of chronic and degenerative diseases, including cancer [143,144]. In addition to the anti-carcinogenic effect, anthocyanins can be used as radiation-protective and chemoprotective agents [145], for the treatment of diabetic retinopathy, fibrocystic disease, and visual impairment [146]. Anthocyanin consumption also reduces capillary fragility and inhibits platelet aggregation [147]. Jasmonates also regulate the accumulation of glucosinolates in some cruciferous species [148]. It has been shown that the regular consumption of cruciferous plants reduces the risk of developing cancer of the stomach, lungs, and intestines [149,150,151], and the anticarcinogenic activity of these products is associated with the presence of glucosinolates [152].

In many experimental works, the direct protective and therapeutic effects of jasmonates on humans and animals have been demonstrated. Since the results of these studies are represented in several review articles and/or are patented [153,154,155], here we only briefly describe the most important results. One of the most studied properties of jasmonates is their therapeutic potential in cancer treatment [156,157,158,159]. Jasmonates inhibit cell proliferation and induce apoptosis or necrosis in various mouse and human cancer cell lines, including breast, prostate, melanoma, lymphoblastic leukemia, and lymphomas [157,160]. Moreover, jasmonates exhibit selective cytotoxicity against cancer cells even in mixed populations of normal and leukemic cells. Methyl jasmonate proved to be a very effective compound, which not only exhibits activity in vitro but also increases the lifespan of mice with lymphoma [157,158,161]. The administration of methyl jasmonate, (Z)-jasmone, and jasmonic acid to the cultured neuroblastoma cell line SH-SY5Y, one of the most common solid tumors in children, leads to a decrease in cell proliferation in a dose- and time-dependent manner, with cancer cells arrested at the G2/M phase [162]. At the same time, the growth of the human embryonic kidney cell line was not affected by jasmonates.

Biological activity has been demonstrated for the biosynthetic precursor of jasmonic acid, 12-OPDA [163]. 12-OPDA can reduce the concentration of free nuclear β-catenin in breast cancer cells [163]. β-catenin plays a key role in the signaling pathway regulated by the growth factor Wnt, which is involved in differentiation, apoptosis, proliferation, and the maintenance of the stem cell pool. Upon the Wnt binding to the cell membrane receptors, β-catenin is transferred from the cytoplasm to the nucleus, where it binds to transcription factors and regulates the genes responsible for cell proliferation. It is also responsible for the degradation of cyclin D1 (overexpression of which leads to the formation of cancerous tumors), leading to cell cycle arrest at the G1 stage [164]. The toxicity of 12-OPDA for humans has not yet been determined.

Several mechanisms of the anti-cancer effects of MeJA have been identified [153]. In one study, it was shown that MeJA treatment leads to the depletion of ATP in cancer cells [165]. Another mechanism is associated with jasmonate-induced de-differentiation of cells through stimulation of the activity of MAPK kinase cascade. This mechanism has been observed in human myeloid leukemia cells, where MeJA and a jasmonic acid derivative, 4,5-didehydrojasmonate, induced the differentiation of leukemia cells and lead to apoptosis [166,167]. In several cell lines, including lung carcinoma cells, jasmonates induce the formation of reactive oxygen species, leading to apoptosis [167]. Jasmonates cause non-apoptotic death in mutant B-lymphoma cells that are highly resistant to radiation and chemotherapeutic drugs [168].

Besides the fact that jasmonates themselves can be used for the treatment of cancer, these oxylipins can be combined with other antitumor agents to achieve a synergistic effect. Many modern chemotherapy treatments use multi-component drugs, which allow the administration of lower doses of substances, reduce undesirable side effects, and even overcome drug resistance [153,160,169].

Metabolites of the jasmonate pathway share structural similarity with animal anti-inflammatory molecules—prostaglandins [156]. This similarity has sparked interest in jasmonates as anti-inflammatory agents. Several studies have confirmed the ability of jasmonates to exert typical prostaglandin anti-inflammatory effects associated with inhibition of the release of inflammatory mediators and alterations in the level of antioxidants [101,170,171]. The anti-inflammatory properties of MeJA, were manifested in mouse macrophages by a decrease in the expression of pro-inflammatory cytokine genes (*IL-1β*, *IL-6*, and *TNF-α*), the suppression of nitric oxide (NO) formation, and inhibition of the NF-κB signaling pathway [170,172]. Methyl jasmonate is considered a promising agent for the treatment of inflammatory bowel diseases—the pathologies characterized by chronic inflammation of the intestines, such as Crohn’s disease and ulcerative colitis [173]. In these diseases, the use of jasmonate leads to a decrease in the expression of tumor necrosis factor and an alteration in the rate of reactive oxygen species formation, which, in turn, leads to a change in the expression of caspase-type protease genes involved in apoptosis. Importantly, this occurs exclusively in disease-carrying cells, not in healthy cells.

By reducing the production of reactive oxygen species in the liver and slowing down systemic inflammation, methyl jasmonate attenuates induced arthritis in Holtzman-source albino rats [174,175], which is characterized by an increased content of reactive oxygen species and a predisposition to the development of an inflammatory reaction [176].

Recently, the anti-inflammatory and antioxidant effects of MeJA on microglial cells, resident macrophages of the central nervous system, have been shown, pointing out the possibility of using jasmonates in the development of new therapeutic approaches for the treatment of Alzheimer’s disease [177]. The anti-neuroinflammatory activity of MeJA was convincingly demonstrated in the lipopolysaccharide-induced inflammation of the mouse brain since MeJA treatment led to a decrease in the level of inflammatory markers—prostaglandin E2, inflammatory cytokines (TNF-α and IL-1β), cyclooxygenase COX2, inducible nitric oxide synthase (iNOS), and NF-κB [178]. Moreover, intraperitoneal administration of MeJA (5–20 mg/kg) helps to reduce brain TNF-α levels in mice exposed to unpredictable chronic mild stress [179].

The ability of the jasmonic acid precursor, 12-OPDA, to regulate inflammatory responses is not surprising, since the chemical structure of OPDA is particularly similar to that of prostaglandins. The influence of OPDA on the course of inflammatory processes was shown, in particular, on microglial cells [180].

Not only natural jasmonates but also synthetic analogs and derivatives, such as methyl dihydrojasmonate or halogenated derivatives, show high biological activity [181,182]. Methyl dihydrojasmonate has been shown to bind to the targets of miR-155 and NF-κB signaling pathways, and 4,5-didehydrojasmonate induces the differentiation of leukemia cells.

Several patents describe the use of jasmonates for the improvement of muscle functions, including the heart muscle (E. A. Bababunmi, US6887499, 2005; B. Broady, US 2012/0172450, 2012) [155].

The positive effect of methyl jasmonate on mental health and the nervous system has been confirmed. MeJA exerted a positive effect on various pathological manifestations such as anxiety, aggression, depression, memory impairment, psychosis, and stress. In connection with these discoveries, the possibility of using methyl jasmonate as a drug for the treatment and prevention of behavioral and neurological disorders was suggested [183]. One of the first reports on this topic showed that MeJA has a calming effect and enhances GABAergic neurotransmission [184]. GABAergic neurotransmission is involved in the physiopathology of Alzheimer’s disease and may serve as a possible target for pharmacological intervention at the early stages of the disease [185]. Also, MeJA reduced the manifestation of rotenone-induced Parkinson-like symptoms in mice, such as a decline in cognitive abilities, depression-like disorders, and postural and motor instability, through the suppression of oxidative stress and inflammation [186]. In mice, MeJA had an antidepressant effect in both acute and chronic stress [179,187]. The MeJA-induced effects, such as reduction of the immobility period in forced swimming or tail suspension tests, are comparable to those of imipramine hydrochloride, a well-known drug for depression treatment. Under unpredictable chronic mild stress conditions, MeJA activates the adaptogenic abilities of the animals, and relieves anxiety and memory impairment [188]. MeJA administered intraperitoneally at concentrations of 1, 5, and 10 mg/kg reduced the symptoms of aggression in a dose-dependent manner [189]. Importantly, the use of this oxylipin did not lead to a decrease in the defense reactions of the body.

The molecular basis of MeJA’s effects on the mental health and nervous system has been little studied so far. It was assumed that the MeJA effects are based on the modulation of the activity of the antioxidant system, neuroprotection, and neuronal regeneration, as well as the regulation of levels of the neurotransmitter, inflammatory biomarkers, and corticosterone [190,191]. Indeed, MeJA reduced oxidative stress, which was seen in lowered malondialdehyde levels and increased glutathione levels in mouse brains under unpredictable chronic mild stress conditions [190]. In addition to the neuroprotective activity associated with a decrease in the oxidative stress level, MeJA suppresses the activity of acetylcholinesterase, responsible for the metabolism of acetylcholine, an important mediator of the central nervous system [171]. It was suggested that MeJA can influence serotonergic and noradrenergic neurotransmitter systems [187,192]. 

The antipsychotic properties of MeJA are confirmed by its effect on psychosis manifestations in mice, such as stereotypic behavior (constant licking, sniffing, chewing, and head movements) [193].

The positive effect of methyl jasmonate on memory has been demonstrated in tests assessing the ability of animals to prevent adverse events using memories from previous experiences [171,183]. MeJA reduces the negative effects of lipopolysaccharides on memory by regulating the expression of the *Aβ(1–42)* gene [171]. Through special tests, it has been proven that MeJA improves spatial memory in mice [171]. This allows us to consider jasmonate as a possible therapeutic agent in Alzheimer’s disease treatment since the mechanisms associated with spatial working memory are noticeably impaired in this disease. Also, MeJA helps to maintain connections in the dendritic network in the dark matter and the striated body of the mouse’s brain, and the cause of Parkinson’s disease is precisely the malfunction of neurons in the dark matter [194]. The therapeutic potential of this substance is enhanced by the fact that MeJA does not affect locomotor functions, exploratory drive, or psychomotor activity [187].

Methyl jasmonate is widely used as a fragrance ingredient in perfumes, cosmetics, shampoos, and soaps [155]. In addition, some jasmonate derivatives, in particular tetrahydrojasmonic acid and (3-hydroxy-2-pentylcyclopentyl)-acetic acid (**4**), have a beneficial rejuvenating effect on human skin, causing extracellular matrix remodeling and improving healing by accelerating the recovery of the epithelium [153,195].

Thus, jasmonates and their derivatives can both directly and indirectly affect the health of humans and animals. It should be noted that most of the studies on the direct effect of jasmonates were carried out on cell lines or model animals, which makes it difficult to apply the results to humans. It is important that some jasmonates, in particular methyl jasmonate and jasmonic acid, are considered safe compounds, and there are no restrictions on their use [196]. Thus, the US Federal Environmental Protection Agency (EPA) has characterized MeJA as a naturally occurring plant hormone that is considered a safe and natural part of the human diet [197]. Several jasmonates and their derivatives have been confirmed to be non-toxic to humans and other non-target organisms in all uses [198]. MeJA has also been evaluated and approved by the Food and Agriculture Organization/World Health Organization (FAO/WHO) as a dietary supplement [199].

### 4.3. Hydroperoxide Lyase Branch Oxylipins

The possibilities of using metabolites from the hydroperoxide lyase branch of the oxylipin biosynthesis pathway in medicine have been little explored. Traumatic acid (**6**), (2(E)-dodecenedioic acid), is the most studied compound of this branch in terms of practical application, although information about the functions of this non-volatile compound in the plant itself is rather limited [200]. As the name suggests, traumatic acid is produced in the plant in response to injury and regulates tissue healing. This compound attracts researchers as a potential wound-healing agent and an intermediate in prostaglandin synthesis through the formation of traumatic lactone [201,202].

Traumatic acid (TA) exhibits a variety of positive effects on normal fibroblasts in vitro, including an antioxidant effect and stimulation of collagen biosynthesis. It has been suggested that TA can be used in preparations for the treatment of skin diseases associated with oxidative stress and collagen biosynthesis and as a substance stimulating mucosal re-epithelialization [203,204]. TA is already used in dental medications such as the gel Restomyl (https://www.buccosante.eu/en/prod/restomyl (accessed on 24 September 2022)).

Interestingly, while exerting an antioxidant effect in normal fibroblasts, TA behaves like a pro-oxidant in cancer cells [203,205,206]. The anticarcinogenic effect of TA manifested itself in a significant dose-dependent reduction in the viability of cancer cells in the three breast cancer cell lines analyzed, while the number of healthy breast epithelial cells increased. The observed decrease in the viability of cancer cells was more pronounced in estrogen-dependent cell lines—MCF-7 and ZR-75-1. TA reduced the viability of these cells by increasing oxidative stress and apoptosis [205,206,207]. The cytotoxic effect of TA on healthy cells was manifested only at high concentrations [207].

Traumatic acid (**6**) was one of the metabolites that noticeably increased in the blood of patients infected with *Plasmodium falciparum*, the protozoan that causes malaria, and it was suggested that this metabolite originates from the pathogen [208]. Although the functions of TA in the parasite remain unknown, it is believed that the biosynthetic branch leading to its formation may be a promising target for the development and optimization of new antimalarial drugs [209].

A study based on computer simulations showed that traumatic acid can be a phytochemical inhibitor of the large (L) polymerase from the dangerous tick-borne bunyavirus Severe fever with thrombocytopenia syndrome virus (SFTSV) due to its ability to bind to the N-terminal endonuclease domain, a target for antiviral drugs [210]. Among the 14,000 studied plant metabolites, traumatic acid turned out to be one of the three most promising candidates, due to the possibility of multiple electrostatic and hydrophobic interactions in the enzyme-TA complex [210].

Volatile compounds of the HPL branch have found wide application in the industry [211]. Hexanal, (Z)-3-hexenal, and (E)-2-hexenal (**7**) are produced on an industrial scale and used as additives to foods for a fresher scent. In addition, these compounds are known to have bactericidal and fungicidal properties, which stipulates their use in the storage of products and cosmetics [211,212,213]. (E)-2-hexenal also shows activity against nematodes [214]. HPL branch compounds exhibit acaricidal activity [215]. Due to the combination of the mentioned properties, these metabolites have found wide application in the production of cosmetics and personal care products [213].

As mentioned above, oxylipins with conjugated double bonds, so-called reactive electrophile species, show high chemical activity. One of the most notable examples is (E)-2-hexenal. It was shown that (E)-2-hexenal can inhibit glutathione S-transferase (GST) activity in melanoma cells [216]. These results are in good agreement with previously published data on the ability of related compounds (E)-2-octenal and (E)-3-nonen-2-one to inhibit the activity of GST isozymes in rat liver [217]. (E)-2-hexenal and related oxylipins containing conjugated double bonds can be considered as a tool to modify the activity of GST isoenzymes that perform a variety of functions in the human body [218].

Ten-carbon aldehydes from diatoms, (2E,4Z,7Z)-2,4,7-decatrienal, (2E,4E,7Z)-2,4,7-decatrienal, and (2E,4E)-2,4-decadienal (**10**), had an antiproliferative effect and stimulated apoptosis in human carcinoma cells [219]. Aldehydes (2E,4E)-2,4-decadienal (**10**), (2E,4E)-2,4-octadienal (**11**), and (2E,4E)-2,4-heptadienal (**12**) had a toxic effect on adenocarcinoma cells of the lungs and rectum without negatively affecting normal cells [220]. The most active compound, (2E,4E)-2,4-decadienal, similarly to the most known anticancer drugs, activated apoptosis of cancer cells, but unlike other known anticancer drugs that promoted an intrinsic cell death pathway, this compound activated the extrinsic (receptor-mediated) apoptotic machinery.

### 4.4. Oxy-, Hydroxy-, and Epoxy-Derivatives of Fatty Acids

Fatty acid hydroxy derivatives are formed in several branches of the oxylipin biosynthesis pathway, both as a result of direct fatty acid oxidation by α-dioxygenase (α-DOX) and as a result of further transformations of fatty acid hydroperoxides formed by lipoxygenases with the participation of reductase, peroxygenase, and epoxyalcohol synthase, including reoxidation of fatty acid hydroperoxides by lipoxygenase. As a result of the epoxy alcohols’ hydrolysis, trihydroxy derivatives of fatty acids can be formed [221], and dihydroxy derivatives can be formed by epoxide hydrolases [222]. Therefore, hydroxy-, dihydroxy-, trihydroxy-, oxo-, epoxy-, or keto-derivatives of fatty acids are widely represented in plant tissues. Nevertheless, there are very few studies on the practically significant properties of these metabolites.

Isomers of 9,10,13-trihydroxy-11-octadecenoic (**15**) and 9,12,13-trihydroxy-10-octadecenoic acids (**16**) isolated from the onion *Allium cepa* have been shown to exhibit prostaglandin-E2-like activity and inhibit platelet aggregation [223]. Most likely, it is these compounds that determine the medicinal properties of onions, which are used in traditional medicine for the treatment of atherosclerosis and gastrointestinal ulcers. Trihydroxy-octadecadienoic acids with prostaglandin-like activity were also isolated from the roots of another medicinal plant, *Bryonia alba* [224].

Dihydroxy triene derivatives formed from docosahexaenoic acid by the double lipoxygenation of soybeans inhibit human blood platelet aggregation at sub-micromolar concentrations and display anti-inflammatory properties [225]. α-Linolenic acid-derived di-hydroxylated metabolites, 9(*S*),16(*S*)-dihydroxy-10E,12Z,14E-octadecatrienoic and 9(*R*),16(*S*)-dihydroxy-10E,12Z,14E-octadecatrienoic acids (**22**), decreased the level of prostaglandins synthesized by recombinant cyclooxygenase COX-1, inhibited platelet aggregation triggered by collagen, and significantly decreased the formation of endogenous oxylipins, leukotriene B4 (LTB4) and 5-hydroxyeicosatetraenoic acid (5(*S*)-HETE), formed from arachidonic acid by 5-LOX of human polymorphonuclear leukocytes [226].

Oxylipin from the roots of *Zanthoxylum zanthoxyloides* (Lam) Zepern. & Timler, 9-oxo-10,12-octadecadienoic acid (**23**), selectively inhibits the in vitro growth of *Trypanosoma brucei,* causing the “sleeping sickness” disease [227]. Treatment with this oxylipin caused significant oxidative stress in *T. brucei* cells, stopped the parasite cell cycle at the G0–G1 transition stage, promoted cell aggregation, and induced morphological changes in parasite cells.

Some hydroxy derivatives of linolenic acid exhibited cytotoxicity against cancer cells [228,229]. An oxylipin-enriched fraction from stinging nettle *Urtica dioica* showed antiproliferative activity against non-small cell lung cancer cells, selectively killing these cells by inducing ER-mediated apoptosis, while not having a toxic effect on normal lung cells [230]. The main active ingredient was identified as 13-S-hydroxy-9Z,11E,15Z-octadecantrienoic acid (**28**). The fatty alcohol ester nonyl 8-acetoxy-6-methyloctanoate (NAMO) (**30**), isolated from the diatom algae *Phaeodactylum tricornutum*, has anticancer effects on three different cancer cell lines, including human leukemia (HL-60), lung carcinoma (A549), and mouse melanoma [231]. (9Z,11E,13S,15Z)-13-hydroxyoctadeca-9,11,15-trienoic acid (13-HOTE) (**28**), a major oxylipin from the microalgae *Chlamydomonas debaryana*, and (5Z,8Z,11Z,13E,15S,17Z)-15-hydroxyeicosa-5,8,11,13,17-pentaenoic acid (15-HEPE) (**29**) from *Nannochloropsis gaditana* displayed a cytotoxic effect against melanoma cells, which was associated with the capability of these compounds to deplete ATP [232]. In addition, the combination of 13-HOTE with the anticancer drug 5-fluorouracil induced synergistic toxicity against colon adenocarcinoma HT-29 cells.

Hydroxy derivatives of fatty acids from the aforementioned microalgae, 13(*S*)-hydroxyoctadecadienoic (**27**) and 13(*S*)-hydroxyoctadecatrienoic (**28**) acids from *Ch. Debaryana,* and 15S-hydroxy-eicosapentaenoic acid (**29**) from *N. gaditana,* demonstrated an anti-inflammatory effect, decreased pro-inflammatory cytokine production in THP-1 macrophages, including IL-1β and IL-6, as well as *iNOS* and *COX-2* expression levels [233]. It was suggested that these oxylipins could be used for the treatment of inflammatory diseases such as inflammatory bowel disease. Hydroxy- and oxy-derivatives of fatty acids from corn and rice also showed a suppressive effect on polysaccharide-induced NO production and expression of several pro-inflammatory genes [234].

Epoxy alcohols can be products of the catalytic action of various enzymes: peroxygenases, lipoxygenases, and epoxyalcohol synthase [43,46,47]. Epoxy alcohols containing double bonds are also formed in animal tissues, mainly from 20-carbon fatty acids, where they perform important regulatory functions [235,236]. There is very little information on the ability of plant epoxy alcohols to influence processes in animal cells. The antimicrobial and fungicidal properties of epoxy alcohols, 9-hydroxy-10,11-epoxy-octadecadienoic, 11,12-epoxy-13-hydroxyoctadecadienoic acids, and trihydroxy acids derived from epoxy alcohols have been described [237]. In living cells, epoxy alcohols can be transformed into hydroxy derivatives [236,238,239], individual representatives of which are described above [25,240].

### 4.5. Phytoprostanes and Phytofurans

Phytoprostanes and phytofurans are products of non-enzymatic oxidation of polyunsaturated fatty acids formed in all plant tissues considered structural analogs of animal isoprostanes and prostanoids [89,90,95,241]. Significant similarities with active regulators of biological processes in the animal cell determine the growing interest in these compounds. In addition to being present in significant amounts in plant foods, they affect human health [241,242,243]. Phytoprostanes, taken orally in vegetable oils, have been found to circulate in plasma in free and conjugated forms, influencing the immune system [244]. However, individual metabolites of this group have received little attention because most studies used the extracts enriched with a mixture of several compounds rather than pure phytoprostanes or phytofurans.

The anti-inflammatory, immunomodulatory, and other health-promoting properties of products containing phytoprostanes have been confirmed in several studies [245,246,247,248]. Given that these compounds are well absorbed by intestinal cells, their health effects should not be underestimated. *Gevuina avellana* nut oil, which exhibits potential health-promoting activities, contains eight phytoprostanes and three phytofurans [247]. An extract from the edible red algae *Gracilaria longissimi* enriched with phytoprostanes and phytofurans affected the pro-inflammatory cytokine gene expression [248]. Olive oil extract enriched with phytoprostanes has demonstrated a hypoglycemic (anti-diabetic) effect [246].

It was previously believed that proteins were the main pollen allergens, but studies have shown that pollen phytoprostanes can also cause allergic reactions [249,250]. The authors suggest that the allergenicity of phytoprostanes is associated with their interaction with T-cells, leading to an increase in the synthesis of the pro-inflammatory cytokine IL-12 and the formation of an allergic reaction [249,251].

Analysis of the effect of phytoprostanes on SH-SY5Y neuroblastoma cells, used as a model of undifferentiated neurons especially sensitive to oxidative stress, showed that phytoprostane B1 increases the metabolic activity of cells, protects against oxidative damage, and promotes differentiation of oligodendrocyte progenitors [245,252]. Phytoprostanes did not affect cells in which the process of differentiation had already begun. The same phytoprostanes acted on immature oligodendrocytes, stimulating their differentiation into mature cells, although they did not show a protective effect under conditions of oxidative stress. The neuroprotective properties and stimulation of nerve fiber myelination are thought to be carried out through via the nuclear receptor PPAR-γ, a ligand-dependent transcription factor that is involved in the control of inflammation, immunity, and cell differentiation [245,253]. The same receptor is involved in the signal transduction of phytoprostane E1 from the pollen of white birch *Betula alba*, which inhibits lipopolysaccharide-induced NF-κB activation and, consequently, pro-inflammatory cytokine (IL-12) synthesis [254].

The ability of several phytoprostanes and phytofurans to modulate inflammatory responses mediated by prostaglandins in lipopolysaccharide-stimulated THP-1 monocytic cells was also demonstrated [255]. It is presumed that the consumption of foods enriched with these oxylipins may have an anti-inflammatory effect.

Phytoprostanes modulate the function of immune cells and exhibit anti-cancer activity in different classes of cancer cells. Phytoprostanes A1, deoxy-PPJ1 (**41**), and, to a lesser extent, B1, exhibit anti-inflammatory activity, induce apoptosis, and modulate the expression of several genes related to the cell cycle in the cells of the leukemic T-lymphocyte line (Jurkat T-cells), and the mechanism of their action is most likely the same as that of endogenous regulators—prostaglandins [241,244]. The efficiency of the induction of malignant cell apoptosis is dependent on the structural organization of the molecule, and this efficiency cannot always be predicted by the structural analogy with prostaglandins. In addition, phytoprostane 16-A1 induces apoptosis of T-cell lymphoma to a greater extent than prostaglandin A2, whereas phytoprostanes 16- and 9-B1 (**42**), structural analogs of the phytoprostane A1, were found to be inactive [244].

Cytotoxicity, chemosensitization, and anti-migratory activities of phytoprostanes and phytofurans were demonstrated on the breast cancer cell lines MCF-7 and MDA-MB-231 [256]. Phytoprostane *Ent*-9-L_1_ reduced the cell viability of both lines, while phytoprostanes 16-F_1t_ and 9-L_1_ (**43**) reduced the cell viability of only one of the two lines. In combination with a subcytotoxic dose of doxorubicin, these phytoprostanes significantly increased the cytotoxic effect on MCF-7 cells, while the chemotherapeutic drug itself had no effect. Phytofuran *Ent*-9-(*RS*)-12-*epi*-ST-∆^10^-13 (**48**) noticeably inhibited the metastatic activity of MDA-MB-231 cells. The possibility of using these compounds as adjuvants to increase the effectiveness of drugs for the treatment of breast cancer has been noted.

### 4.6. Unusual and Unidentified Oxylipins

**Figure 8:**
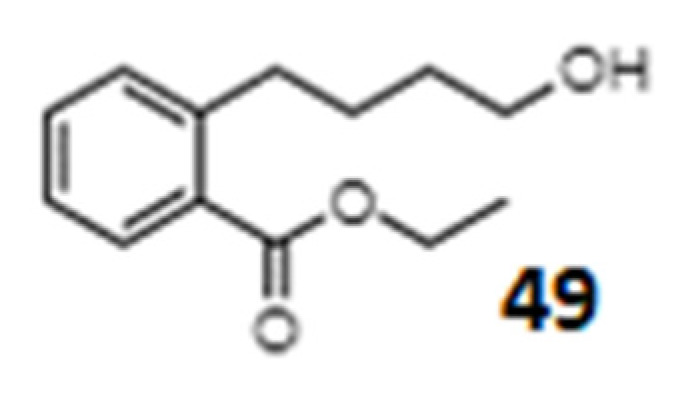
Chemical structures of a rare complex oxylipin momordicatin, 4-(o-carboethoxyphenyl) butanol (**49**).

In addition to the information about the mentioned classes of compounds, there are examples of the analysis of individual oxylipins with a more complex chemical structure (Figure 8). Momordicatin, 4-(o-carboethoxyphenyl) butanol (**49**), from *Momordica charantia* fruit, was effective in vitro and in vivo against *Leishmania donovani* [257]. It inhibited the parasite’s iron-containing superoxide dismutase (SOD) without affecting the host’s SOD.

There are also multiple examples of studies where the effects caused by the extracts containing unidentified oxylipins were described, and the active components of these extracts have yet to be identified. For example, the anti-inflammatory effect of the lyophilized biomass of microalgae *Chlamydomonas debaryana* enriched with oxylipins was demonstrated on a mouse colitis model [258]. Unidentified diatom oxylipins exhibit antibacterial, anti-parasitic, anti-inflammatory, and anti-cancer properties [259]. Ethyl extracts and butanol fractions isolated from *Tinospora sinensis* induced an oxidative burst in macrophages by increasing the production of ROS and NO, which led to the destruction of *Leishmania donovani* [260].

## 5. Conclusions

Thus, an impressive number of studies confirm the ability of plant oxylipins to influence the various processes in animal cells and their protective and therapeutic properties (Table 1). At the same time, the potential of many oxylipins has not been determined until now. It primarily applies to many phytoprostanes, oxy-, epoxy-, and hydroxy-derivatives of fatty acids. Further progress in this research area and the application of plant oxylipins in medical practice depends on the interdisciplinary research at the interface between plant biology and medicine dedicated to the search for new natural metabolites, the evaluation of their therapeutic potential, and the creation of synthetic analogs with improved properties, such as increased activity, stability, and the ability to reach intracellular targets.

## Figures and Tables

**Table 1:** Potential therapeutic and protective properties of plant oxylipins.

Oxylipin	Concentration	The Studied System	Effect	Refs.
Derivatives of acetylenic fatty acids				
Falcarinol (**31**)	0.5–20 μM	Tissue culture, mouse model	Stimulates neuritoge-nesis, restores memory mechanisms, neuropro-tective properties	[134,135]
	0.016–2 μg/mL	Human gastric adenocarcinoma, leukemia like, mouse fibroblast-derived tumor, mouse melanoma	Anti-cancer properties	[80]
	0.1–10 μM	Transfected cells of human embryonic kidney	reversible agonist of cannabinoid receptors	[261]
Falcarinol (**31**) and didehydrofalcarinol (**32**)	20–200 g/mL	Fungal spores	Antifungal properties	[82,118]
Falcarinol (**31**), falcarindiol (**33**) and falcarindiol-3-acetate (**34**)	0.5–20 μM	Mice macrophages cell lines	Anti-inflammatory, NO production inhibition, no cytotoxicity	[131]
	100 μg/m1	Rabbit blood	Antiplatelet effect	[113]
Falcarindiol (**33**), falcarinol (**31**)	10 μg/mL MIC 16.4 μM,	In vitro activity	Antimicrobe and antimycobacterial	[119,120,121,122]
Falcarindiol-8-methyl ether (**35**); panaxydiol (**40**)	IC50 3.5 μM/l	Human cancer cell lines	Cytotoxic effect	[80,124]
Furanocoumarin ethers of falcarindiol (**38**)	ED50, 3.2–8.5 μg/mL	Tissue culture	Antiproliferative activity	[125]
11(*S*),16(*R*)-Dihydroxy-octadeca-9Z,17-dien-12,14-diyn-1-yl acetate (**39**); falcarindiol (**33**)	IC50 24 μM; 73 μM,	In vitro activity	Inhibition of 5-, 12-, and 15-lipoxygenases, cyclooxygenase (COX-1)	[83,128,129]
11(*S*),16(*R*)-Dihydroxy-octadeca-9Z,17-dien-12,14-diyn-1-yl acetate (**39**); (3*R*, 8*S*)-falcarindiol (**33**)	IC50 118 μM	In vitro radiolabeled ligand binding	Serotonin receptor binding, serotonergic, antidepressant potential	[136]
1,2-Dihydro-11-acetoxy-falcarindiol (**36**); 11-Acetoxy-falcarindiol (**37**)	0.001–100 μM; IC50 0.3–29 μM	Human cancer cell lines	Inhibition of cell proliferation	[127]
**Jasmonates**				
JA, 3-oxo-2-(2-pentenyl) cyclopentaneacetic acid (**1**); MeJA (**3**)	0.5–3 µM	Human T lymphoblastic leukemia, breast carcinoma, melanoma, androgen-responsive prostate adenocarcinoma cells; mouse T lymphoma cells	Cell death and inhibition of cell proliferation in cancer cells, no damage to normal lymphocytes	[157]
MeJA (**3**)	1–5 mM	Human carcinoma cell lines	Cell death and growth inhibition	[161]
	20 µM	Microglial cell line BV-2	Protects against β-amyloid-induced oxidative stress and inflammation	[177]
MeJA analogs	12.5–100 µM	RAW264.7 murine macrophage cells	Inhibition of biosynthesis of pro-inflammatory mediators	[170,181]
Jasmonate derivative, (3-hydroxy-2-pentylcyclopentyl)-acetic acid (**4**)	10 µM	Epidermal primary keratinocytes and reconstituted skin epidermis	Induce expression of major skin proteoglycans, skin healing, accelerated epithelial repair *in vivo*	[195]
12-oxo-phytodienoic acid (**2**)	7.5–30 μM	Mouse microglial cells	Suppression of LPS-induced expression of the inflammatory cytokines and NO production	[180]
**Hydroperoxide lyase branch oxylipins**				
Traumatic acid (**6**)	1–10 µM	Fibroblast cell line	Antioxidant and stimulatory effects on collagen biosynthesis	[203]
	0.5–1000 µM	ZR-75-1 cell line, treated with mesotrione to enhance growth	Anticancer activity	[205]
	0.5–1000 µM	Breast cancer cell lines and normal breast cell lines	Reduction of pesticide-induced cancer cell division	[207]
(E)-2-hexenal (**7**); (2E,6Z)-2,6-nonadienal (**8**); (E)-2-nonenal (**9**)	4–314 mg/g	Mites *Acarus siro* L., *Tyrophagus putrescentiae* (Schrank), *Aleuroglyphus ovatus*	Growth inhibition (4–35 mg/g), death (36–314) mg/g	[215]
(2E,4E)-2,4-decadienal (**10**); (2E,4E)-2,4-octadienal (**11**); (2E,4E)-2,4-heptadienal (**12**)	2.5–10 µM	Lung and colon cancer cell lines; normal lung/brunch epithelial cell line	Cytotoxic effect against cancerous but not normal cells	[220]
**Oxy-, hydroxy-, and epoxy-derivatives of fatty acids**				
9,10,13-trihydroxy-11-octadecenoic (**15**); 9,12,13-trihydroxy-l0-octadecenoic acid (**16**)	-	Rabbit coeliac and mesenteric arteries; rat fundus strip; cascade superfusion system	Prostaglandin-like activity–smooth muscle relaxation	[223]
9,12,13-trihydroxy-15-octadecadienoic (**17**); 12,15,16-trihydroxy-9,13-octadecadienoic (**18**); 9,10,13-trihydroxy-11,15-octadecadienoic (**19**); 12,13,16-trihydroxy-9,14-octadecadienoic acid (**20**)	-	Rat colon, suspended strip	Prostaglandin-like activity–smooth muscle relaxation	[224]
10,17-dihydroxy-docosahexa-4,7,11,13,15,19-enoic acid (**21**)	0.3-10 µM	Platelet suspension	Inhibited collagen-induced platelet aggregation in a dose-dependent manner.	[225]
9,16-dihydroxy-10,12,14-octadecatrienoic acid, isomers (**22**)	1 µM	Platelet suspensions, leukocyte suspensions, recombinant COX protein	Anti-inflammatory, antithrombotic effects, inhibition COX-1	[226]
9-oxo-10, 12-octadecadienoic acid (**23**)	EC50 1.2 µM	Blood stream *T. brucei* form, mouse macrophages	*T. brucei* growth inhibition	[227]
13-hydroxy-10-oxo-11-octadecenoic acid (**24**); 10-oxo-11-octadecen-13-olide, enantiomers (**25**)	-	Mouse leukemia cells	Cytotoxicity	[229]
9-hydroxy-10,12-octadecadienoic acid (**26**) 13-hydroxy-9,11-octadecadienoic acid (**27**)	-	Mouse fibroblast cells, simian virus 40-transformed cells	Cytotoxicity	[229]
9-hydroxy-10,12-octadecadienoic acid; 13-hydroxy-10-oxo-11-octadecenoic acid; 10-oxo-11-octadecen-13-olide	0.8–100 µM	Murine macrophages, monkey kidney cells	Anti-inflammatory	[234]
13-hydroxyoctadeca-9,11,15-trienoic acid (**28**); 15-hydroxyeicosa-5,8,11,13,17-pentaenoic acid (**29**)	10 mM	Human colonic adenocarcinoma and melanoma cell lines	Cytotoxicity	[232]
Nonyl 8-acetoxy-6-methyloctanoate (**30**)	25, 50 mg/mL	Human leukemia cells; lung carcinoma; mouse melanoma	Anticancer effects	[231]
13-hydroxy-9,11,15-octadecantrienoic acid (**28**)	-	Non-small cell lung cancer	Anti-proliferative activity	[230]
**Phytoprostanes, phytofurans**				
B1-Phytoprostanes	0.1–25 µM	Undifferentiated neuroblastoma cells	Neuroprotective activity	[245,252]
E1-Phytoprostanes	-	Culture of monocyte-derived dendritic cells	Anti-inflammatory activity	[249,254]
Phytoprostanes A1, E1, and deoxy-J1 (**41**)	10–80 µM	Healthy males ages 18–35 years, human embryonic kidney cells, macrophage-like cells	Anti-inflammatory, apoptosis-inducing activity	[244]
Phytoprostanes from olive oil	-	In vitro inhibition of α-glucosidase and α-amylase	Antidiabetic activity	[246]
16-B-1 (**42**)- and 9-L1-phytoprostanes (**43**)	-	Human neuroblastoma cells	Antioxidant activity	[245]
Phytoprostanes: 9-F1t, 9-epi-9-F1t (**44**), ent-16-F1t, ent-16-epi-16-F1t (**45**), 9-D1t, 9-epi-9-D1t (**46**), 16-B1 (**42**), and 9-L1 (**43**); Phytofurans: ent-16(*RS*)-9-epi-STΔ14-10, ent-9(*RS*)-12-epi-ST-Δ10-13 (**48**), and ent-16(*RS*)-13-epi-ST-9-Δ14-9	-	Human colorectal adenocarcinoma and human endothelial cell lines	Anti-inflammatory	[248]
*Betulla alba* pollen phytoprostane		Monocyte-derived dendritic cells, T cells	Modulation of human dendritic cells function	[249]
Phytoprostanes: 16-F1t, 16-epi-16-F1t (**45**), 16-B1, Ent-16-B1 (**42**), 9-L1, Ent-9-L1 (**43**), 9-E1; Phytofurans: Ent-9-(*RS*)-12-epi-ST-9-∆10-13 (**48**)	0.1–100 µM	Human breast cancer cell lines	Anticancer effects	[256]
Phytoprostanes: 9-F1t, 9-epi-9-F1t (**44**), ent-16-F1t, ent-16-epi-16-F1t (**45**), 9-D1t, 9-epi-9-D1t (**46**), 16-B1 (**42**), 9-L1 (**43**); Phytofurans: ent-16(*RS*)-9-epi-STΔ14-10, ent-9(*RS*)-12-epi-ST-Δ10-13 **(48),** ent-16(*RS*)-13-epi-ST-Δ14-9 **(47)**	0.002–100 µM	The monocytic human (THP-1) cell line	Anti-inflammatory activity	[255]
**Unusual complex oxylipins**				
Momordicatin (**49**) 4-(o-carboethoxyphenyl) butanol	-	*L. donovani* strain	Antileishmania agent	[257]

MIC—Minimal inhibitory concentration; IC50—half maximal inhibitory concentration; ED50—median effective dose.

## Data Availability

Not applicable.

## Abbreviations

LOX	Lipoxygenase
α-DOG	α-Dioxygenase
AOS	Allene oxide synthase
HPL	Hydroperoxide lyase
DES	Divinyl ether synthase
EAS	Epoxy alcohol synthase
JA	Jasmonic acid
MeJA	Methyl jasmonate
12-OPDA	12-Oxo-phytodienoic acid
dn-OPDA	Dinor-12-oxophytodienoic acid
PP	Phytoprostane
RES	Reactive electrophile species
ILs	Interleukins
TNF-α	Tumor necrosis factor alpha
PKC-β	Protein kinase C-beta
NF-κB	Nuclear factor kappa B
PPAR	Peroxisome proliferation activator receptor
TA	Traumatic acid
KEAP1	Kelch-like ECH-associated protein 1
NRF2	Nuclear factor associated with erythroid factor 2
iNOS	Inducible nitric oxide synthase
GABA	γ-Aminobutyric acid
SOD	Superoxide dismutase
